# Musculoskeletal imaging findings of xylazine-associated wounds

**DOI:** 10.1007/s10140-026-02480-4

**Published:** 2026-04-28

**Authors:** Alvaro A. Ordonez, John D. Karp, Peter Qiao, Chantal Chahine, Waqas Bari, Nogah Shabshin

**Affiliations:** 1 Department of Radiology, Hospital of the University of Pennsylvania, Philadelphia, PA USA; 2 Department of Radiology, Emek Medical Center, Clalit Health Services, Afula, Israel

**Keywords:** Xylazine, Osteomyelitis, Bone, Infection

## Abstract

**Purpose:**

Xylazine is a veterinary sedative increasingly detected as an adulterant in illicit opioids that is associated with severe skin and soft-tissue wounds. This study aimed to characterize the musculoskeletal imaging manifestations of xylazine-associated tissue injury in individuals with recreational use and to increase radiologic awareness of this emerging public health problem.

**Methods:**

This institutional review board–approved retrospective study identified patients with documented recreational xylazine use who underwent musculoskeletal imaging. Demographic data, wound location, clinical characteristics, and photographic documentation were extracted from the electronic medical record and correlated with findings on radiography, CT, MRI, and ultrasound.

**Results:**

A total of 120 patients were included. Wounds most commonly involved the lower (48%) and upper extremities (48%). Radiographs were obtained in 90% of patients, CT in 62%, MRI in 16%, and ultrasound in 13%. Radiographs frequently demonstrated distortion of soft-tissue planes with prominent periosteal reaction. CT and MRI revealed extensive soft-tissue hypertrophy with ulceration and necrosis of variable depth, often extending to subcutaneous tissues and underlying muscle. Ultrasound was of limited diagnostic value, primarily for assessing wound extent and detecting abscesses. Advanced cases often showed circumferential involvement of an entire limb segment. Acute osteomyelitis was present in 16% and chronic osteomyelitis in 13%, with osseous involvement limited relative to the severity of soft-tissue injury.

**Conclusion:**

Xylazine-associated wounds demonstrate a distinctive imaging pattern of extensive soft-tissue necrosis and ulceration with marked periosteal reaction but relatively infrequent osteomyelitis. Radiologic evaluation, particularly with radiography and CT, is essential for early recognition, delineation of disease extent, and avoidance of overdiagnosis of osteomyelitis.

**Graphical Abstract:**

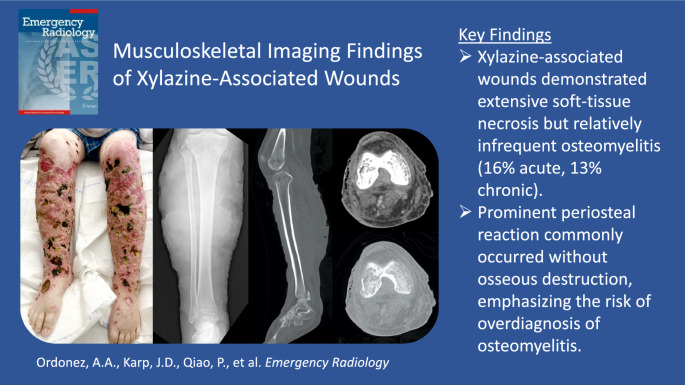

**Supplementary Information:**

The online version contains supplementary material available at 10.1007/s10140-026-02480-4.

## Introduction

Xylazine is a veterinary sedative and analgesic that has emerged as a significant public health concern due to its increasing detection as an adulterant in illicit drugs, particularly in combination with opioids such as fentanyl and heroin [1]. Its low cost, availability, and synergistic sedative effects with opioids [2] have facilitated its widespread incorporation into the illicit drug supply in North America, with growing reports of distribution worldwide [3, 4]. In Philadelphia, for example, xylazine has been detected in up to 98% of fentanyl samples and in 78% of patients with fentanyl-positive urine toxicology [5, 6]. Originally synthesized in 1962 for its potential antihypertensive properties, xylazine acts as an α2-adrenergic receptor agonist that produces profound sedation, muscle relaxation, and analgesia [7]. However, early human trials revealed severe adverse effects, including hypotension, bradycardia, and central nervous system depression, leading to the abandonment of its development for human use [8].

The increasing prevalence of xylazine in the illicit drug supply has introduced new clinical and public health challenges. In cases of opioid overdose, the presence of xylazine complicates management because its depressant effects are not reversed by standard opioid antagonists such as naloxone. As a result, sedation may persist despite naloxone administration, increasing the risk of prolonged respiratory compromise [9, 10]. Beyond its systemic effects, chronic use, especially through repeated subcutaneous or intravenous injection, is associated with a distinct pattern of skin and soft-tissue injury [11, 12]. Xylazine-related wounds often develop at or distal to injection sites but may also occur at locations distant from vascular access points. These lesions typically demonstrate full-thickness skin necrosis, ulceration, and surrounding induration, frequently complicated by secondary bacterial infection [11, 13, 14].

Of particular concern are the musculoskeletal complications that may develop in association with these wounds. The combination of direct tissue toxicity, vasoconstriction-related ischemia, repeated injection trauma, and delayed presentation due to sedation increases the risk of cellulitis, abscess formation, septic tenosynovitis, septic arthritis, osteomyelitis, and pathologic fractures. These conditions can threaten limb viability and often require advanced imaging to determine the extent of disease and guide appropriate medical or surgical management.

Radiologic evaluation plays a critical role in the management of xylazine-associated musculoskeletal complications [15]. Imaging modalities such as plain radiography, computed tomography (CT), magnetic resonance imaging (MRI), and ultrasound provide complementary diagnostic information, ranging from detecting soft-tissue gas and retained foreign bodies to assessing marrow abnormalities and joint involvement. Recognition of the imaging patterns associated with xylazine-related wounds is essential for timely diagnosis, appropriate antimicrobial therapy, and surgical planning.

In this study, we characterize the musculoskeletal manifestations of xylazine-associated wounds in a large cohort of patients from a high-burden urban setting [16], with particular emphasis on their radiologic features. Our aim is to increase awareness among radiologists, emergency physicians, surgeons, and other clinicians by illustrating the spectrum of musculoskeletal imaging findings associated with this rapidly emerging public health crisis.

## Materials and methods

Approval of this study was obtained from the Institutional Review Board (approval #855841), and HIPAA requirements were followed. Patients were identified from a retrospective search of our institutional database. Due to the retrospective nature of the study, informed consent was not required. The search encompassed a review of radiology reports from January 2024 to June 2025. Only patients for whom imaging was available and recreational xylazine use was confirmed were included in the study. Confirmation of xylazine use was based on documentation in the medical record, including patient self-report and toxicology results when available. Available medical records were reviewed for patient demographic information, including age, sex, wound location, and clinical presentation. Imaging studies included radiographs, CT, MRI, and ultrasound. All imaging studies were interpreted in routine clinical practice by board-certified radiologists with musculoskeletal subspecialty training. Osteomyelitis was diagnosed based on imaging features interpreted by musculoskeletal radiologists, with cases categorized as acute, chronic, indeterminate, or negative based on overall imaging appearance. Periosteal reaction alone was not considered diagnostic of osteomyelitis. Imaging was considered highly suspicious for osteomyelitis only when one or more of the following were present: cortical/permeative osseous destruction, intramedullary bone involvement on cross-sectional imaging, soft-tissue gas tracking to bone, or exposed bone with imaging findings consistent with contiguous osseous involvement. In the absence of these features, cases were categorized as negative or indeterminate for osteomyelitis even when periosteal reaction was extensive. When prior or follow-up imaging was available, interval progression of osseous destruction was used as supportive evidence for osteomyelitis.

## Results

A total of 120 patients who presented to the emergency department with musculoskeletal injuries related to recreational xylazine use were included. The median age was 38 years (range, 23–66 years), and 35% of patients (*n* = 42) were female (Table 1). Most patients were White (86%). Wounds most commonly involved legs (41%) and arms or shoulders (35%), corresponding to injection sites. The remaining wounds involved the hands (13%), feet (7%), head and neck (5%), and other locations (2%). At the time of presentation, radiographs were obtained in 90% of cases, while CT and MRI were performed in 62% and 16% of patients, respectively (Table 2). Blood cultures were positive in 13 patients, and leukocytosis was present in 23% of cases, with a mean white blood cell count of 9.3 ± 4.4 × 10⁹/L. Acute osteomyelitis was reported in 16% of patients, chronic osteomyelitis in 13%, and no evidence of osteomyelitis in 61%. In 10% of patients, imaging findings were indeterminate for osteomyelitis. Among patients with positive blood cultures, 38% were diagnosed with acute osteomyelitis.

**Table 1:** Demographic and disease characteristics of the study participants

Characteristic	No. (%)
Age (years)	
<30	6 (5%)
30–39	66 (55%)
40–49	39 (33%)
≥ 50	9 (8%)
Sex	
Female	42 (35%)
Male	78 (65%)
Race and ethnicity	
Black or African American	5 (4%)
Hispanic or Latino	12 (10%)
White	103 (86%)
Location of wounds	
Head and neck	6 (5%)
Hands	15 (13%)
Arms and shoulders	42 (35%)
Legs	49 (41%)
Feet	8 (7%)
Other	2 (2%)
Blood culture	
Positive	13 (11%)
Negative	72 (60%)
Not available	35 (29%)

**Table 2:** Imaging characteristics and diagnosis

Characteristic	No. (%)
Type of imaging obtained	
Radiograph	108 (90%)
CT	74 (62%)
MRI	19 (16%)
Ultrasound	15 (13%)
Diagnosis	
Acute osteomyelitis	19 (16%)
Chronic osteomyelitis	16 (13%)
Indeterminate findings of osteomyelitis	12 (10%)
No osteomyelitis	73 (61%)

### Skin, subcutaneous fat, and deep fascial planes

**Fig. 1:**
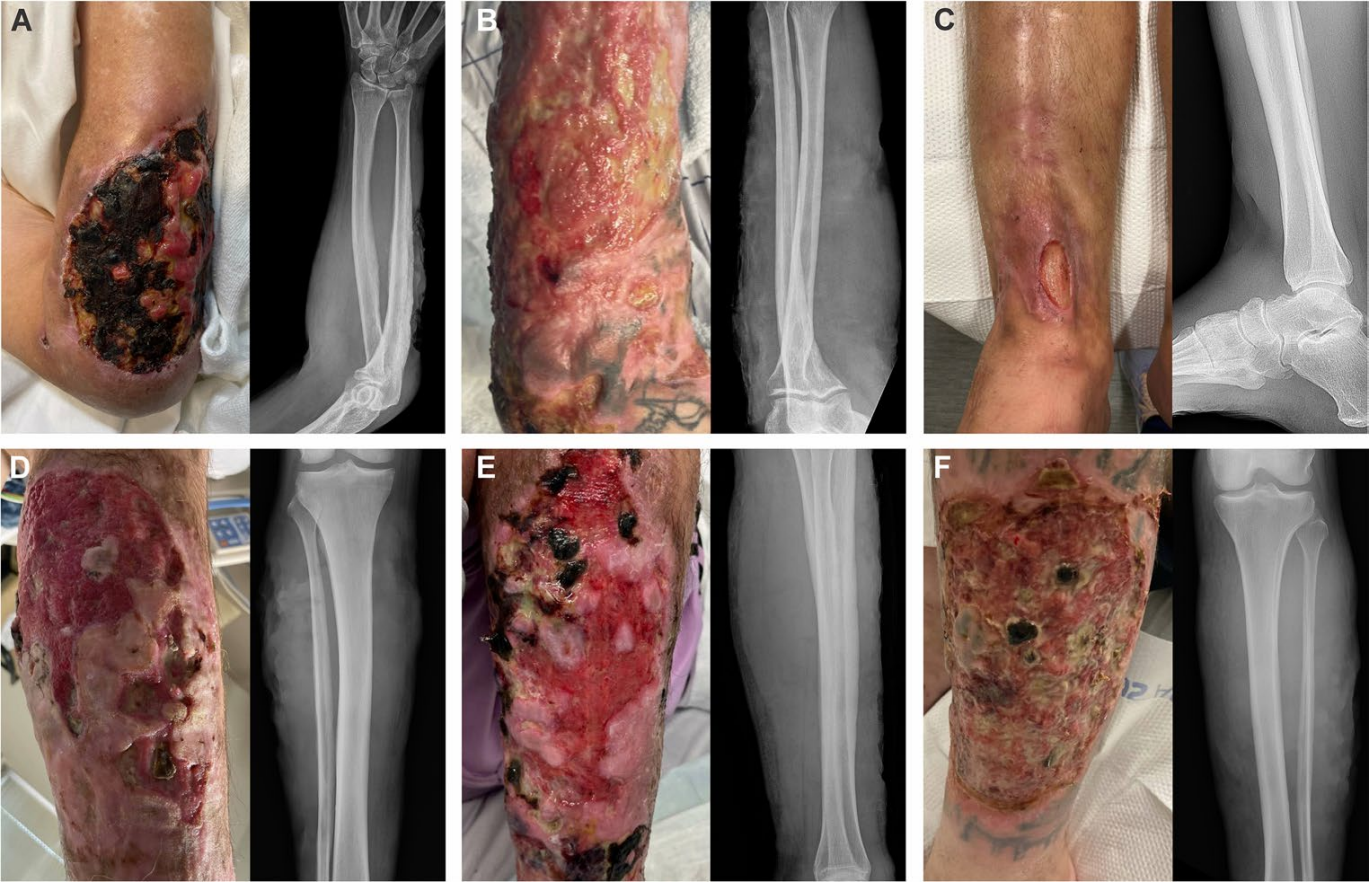
Representative clinical photographs and corresponding radiographs from six patients with xylazine-associated extremity wounds demonstrate marked irregular soft-tissue defects with ulcerated skin, undulating contours, and severe soft-tissue distortion, often disproportionate to the limited osseous findings on radiographs. (**A**) 56-year-old woman with an extensive forearm ulcer and overlying soft-tissue distortion; radiographs demonstrate cortical thickening of the ulna and nonspecific radial cortical thickening. (**B**) 39-year-old man with multiple ulcerated forearm wounds and marked skin irregularity; radiographs demonstrate mild periosteal thickening of the ulna and distal radius. (**C**) 43-year-old man with focal ulceration and surrounding subcutaneous edema of the lower leg; radiographs demonstrate a skin defect without cortical destruction or periosteal reaction. (**D**) 32-year-old woman with diffuse circumferential ulceration of the lower leg; radiographs demonstrate skin defects and limited periosteal reaction of the fibula. (**E**) 49-year-old woman with extensive soft-tissue swelling and irregular ulceration of the lower leg; radiographs demonstrate mild distal fibular cortical irregularity. (**F**) 46-year-old man with large lateral and posterior calf ulcers and severe soft-tissue distortion; radiographs demonstrate bilateral fibular periostitis without extensive cortical destruction

**Fig. 2:**
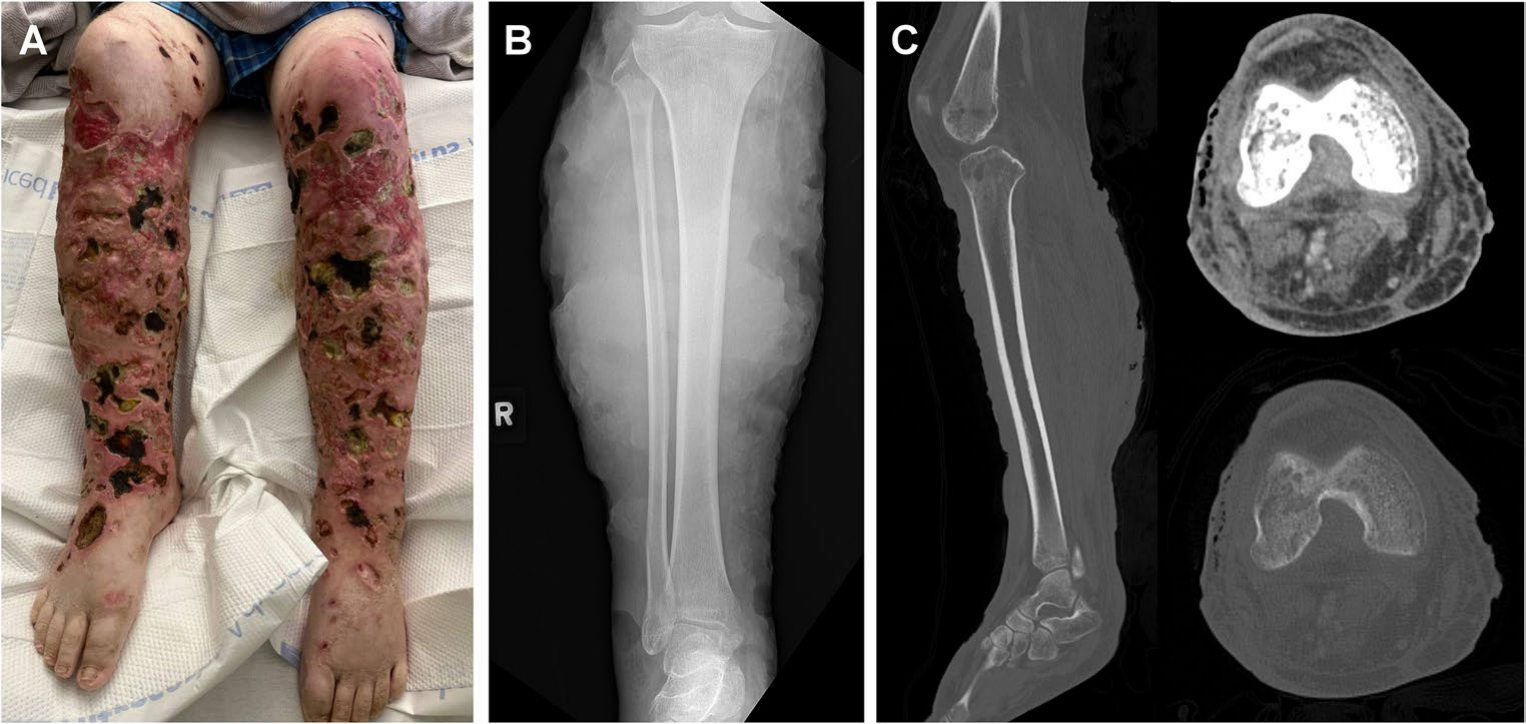
33-year-old man with (**A**) extensive soft-tissue wounds involving both lower extremities. (**B**) Radiographs of the right tibia and fibula demonstrate soft-tissue defects and subcutaneous air. (**C**) Contrast-enhanced CT of the right lower leg shows no gas or ulceration adjacent to the bone, no exposed bone, and no osseous erosion to indicate acute osteomyelitis

**Fig. 3:**
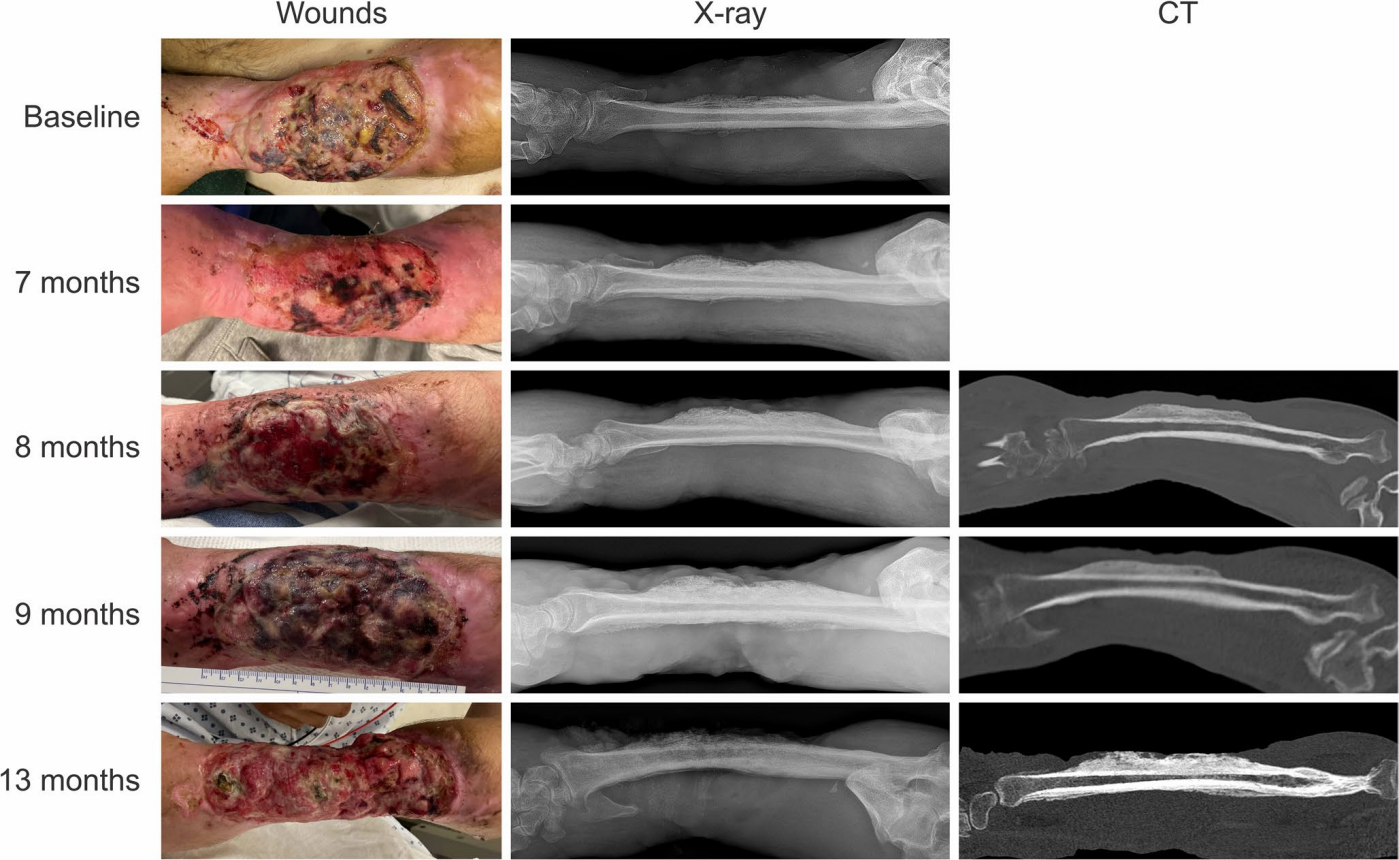
Longitudinal imaging in a 27-year-old man with an extensive soft-tissue wound of the right upper extremity associated with repeated xylazine injections. At baseline, radiographs demonstrated soft-tissue ulceration and periosteal reaction of the radial diaphysis, with segmental resection of the ulna involving the proximal and mid-diaphysis. Follow-up imaging demonstrated progressive radial periosteal reaction, with eventual exposure of the radial diaphysis through the overlying soft-tissue wound 13 months later, consistent with osteomyelitis

Early lesions most commonly manifested as superficial ulcers at sites of repeated xylazine injections. These lesions frequently progressed to full-thickness skin and subcutaneous fat loss with exposure of underlying muscle and tendons (Fig. 1). The ulcers were typically large, heterogeneous in appearance, and localized rather than systemic, with central necrosis and irregular margins resembling chemical or ischemic tissue injury. At this stage, radiographs and CT revealed irregular soft-tissue defects corresponding to ulcerated skin wounds, often with undulating contours and marked distortion of the soft-tissue surface (Figs. 1 and 2). MRI demonstrated skin thickening, superficial inflammatory changes, and associated enhancement. Unlike conventional bacterial soft-tissue infections, xylazine-associated wounds frequently lacked purulence and were often confined to injection sites, although additional lesions could occur within the same extremity segment. In several patients with longitudinal imaging, progressive enlargement of the skin defect was observed over time, reflecting ongoing spreading necrosis rather than rapid contiguous spread (Fig. 3).

**Fig. 4:**
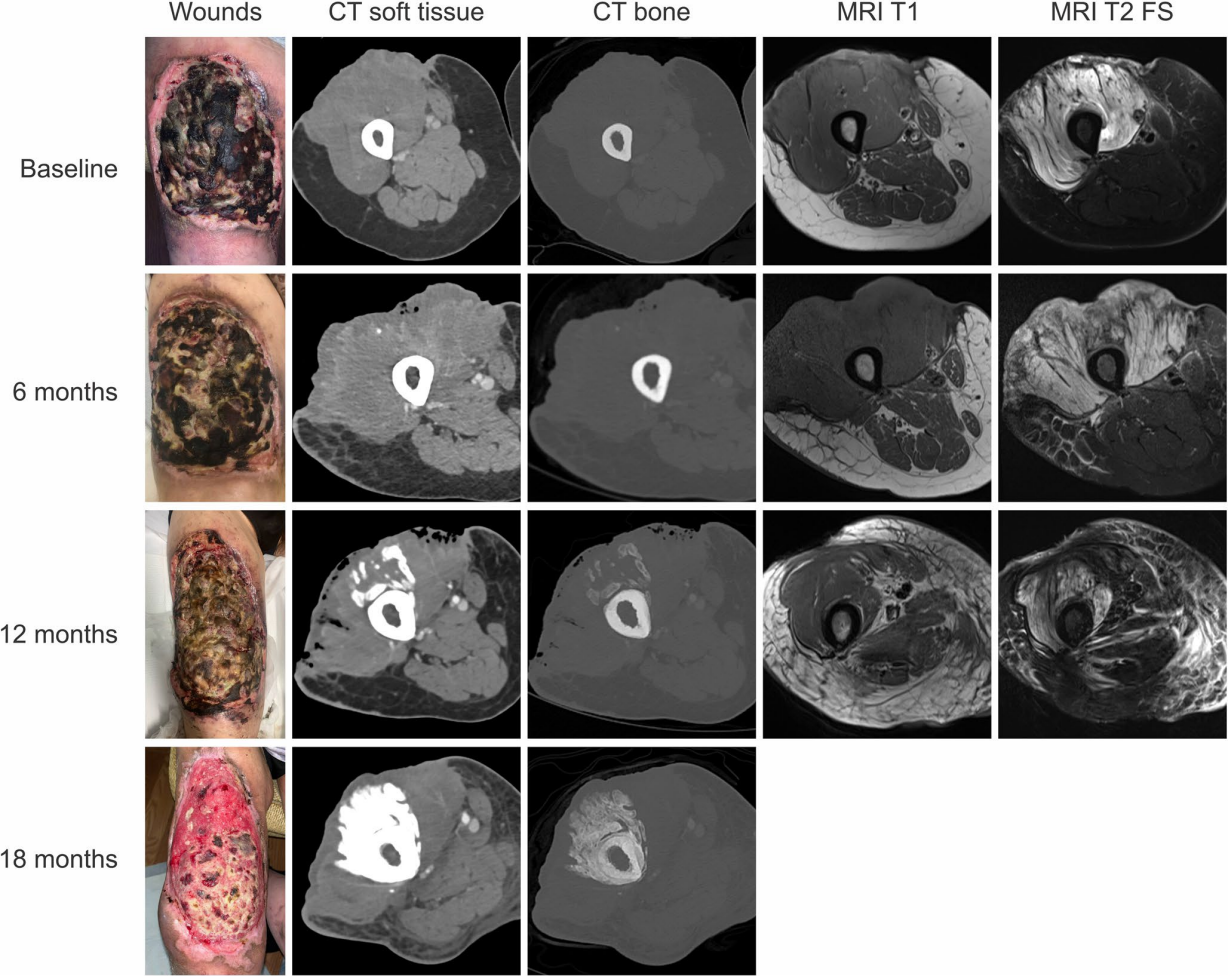
Sequential CT and MRI of the right thigh in a 33-year-old woman with a large anterior soft-tissue wound, obtained at baseline and at 6-, 12-, and 18-month follow-up. At baseline, CT and MRI demonstrate a large anterior proximal-to-mid thigh soft-tissue defect with involvement of the underlying musculature, loss of overlying subcutaneous fat, and associated muscle hypertrophy. No evidence of acute osteomyelitis is present. At 6 and 12 months, CT and MRI demonstrate progressive muscle loss and near-complete absence of subcutaneous tissue, with increasing periosteal reaction on MRI and cortical thickening on CT, consistent with reactive periostitis. Mildly heterogeneous marrow signal within the femoral diaphysis is also present, most compatible with osteitis without imaging evidence of acute osteomyelitis. At 18 months, CT demonstrates further progression of periosteal reaction with cortical thickening and heterotopic ossification along the femoral diaphysis without cortical destruction. This case illustrates extensive periosteal reaction and low T1 marrow signal in the absence of destructive osseous change, emphasizing that these findings may represent reactive periostitis and osteitis rather than osteomyelitis in xylazine-associated wounds

A hallmark imaging feature of advanced cases across modalities was extensive obliteration of subcutaneous fat along long segments, often extending beyond the injection site and typically involving an entire limb segment between two adjacent joints. On CT and MRI, this appeared as near-complete loss of subcutaneous fat planes with replacement by heterogeneous inflammatory or necrotic tissue (Fig. 4).

Imaging also demonstrated diffuse soft-tissue thickening with loss of normal fat lucency, visible as undulation of the fat plane on radiographs and CT. Gas within the soft tissues was present in a subset of patients and was detected on all imaging modalities. When present, gas was typically localized to the ulcer bed or adjacent necrotic tissue rather than tracking along fascial planes, a pattern that distinguished these wounds from other musculoskeletal conditions such as diabetic foot infection, trauma, and necrotizing fasciitis. On ultrasound, these abnormalities manifested as heterogeneous hypoechoic edematous subcutaneous tissue with ill-defined margins and granulation tissue (Supplementary Fig. 1). The role of ultrasound in this setting was limited, primarily serving to assess wound dimensions, identify abscesses, detect retained needle fragments, and monitor interval healing.

Deep fascial thickening and disruption were also observed, with involvement of underlying muscle compartments and, in some cases, tendon sheaths. Muscle abnormalities initially manifested as enlargement and hypertrophy with diffuse edema and T2 hyperintensity on MRI. In patients with longitudinal imaging, a characteristic evolution was observed, with progressive loss of muscle tissue over time, suggesting progression from inflammatory enlargement to devitalization and resorption (Fig. 4; Supplementary Figs. 2 and 3). Devitalized tissue was identified on MRI as areas of non-enhancement on post-contrast imaging. Abscess formation occurred in only a subset of patients and developed within deep soft tissues or adjacent tendon compartments. Less common findings included septic bursitis (Supplementary Fig. 4), tenosynovitis with compartment-level involvement (Supplementary Fig. 5), and localized muscle abscesses at injection sites in the neck (Supplementary Fig. 6).

### Bone involvement: periosteal reaction, reactive osteitis, and osteomyelitis

Reactive osseous changes were common, although true osteomyelitis was relatively infrequent compared with the severity of the overlying soft-tissue injury (Fig. 2). The most striking and recurrent osseous finding across imaging modalities was florid periosteal reaction involving long bone segments, often extending over a larger anatomic region than the cutaneous ulcer and not necessarily centered at the site of skin breakdown. On radiographs and CT, periosteal reaction was frequently lamellated or irregular and occasionally progressed to cortical thickening or heterotopic ossification (Fig. 3). MRI, though performed in a smaller subset of patients, typically showed adjacent reactive marrow edema and periostitis without evidence of marrow replacement. Importantly, in most cases, this periosteal reaction occurred without imaging findings to support contiguous osteomyelitis, including absence of low T1 marrow signal, cortical destruction, gas extending to bone, or exposed bone. These findings were consistent with reactive osteitis or periostitis, reflecting an inflammatory response to adjacent soft-tissue injury rather than a true bone infection.

**Fig. 5:**
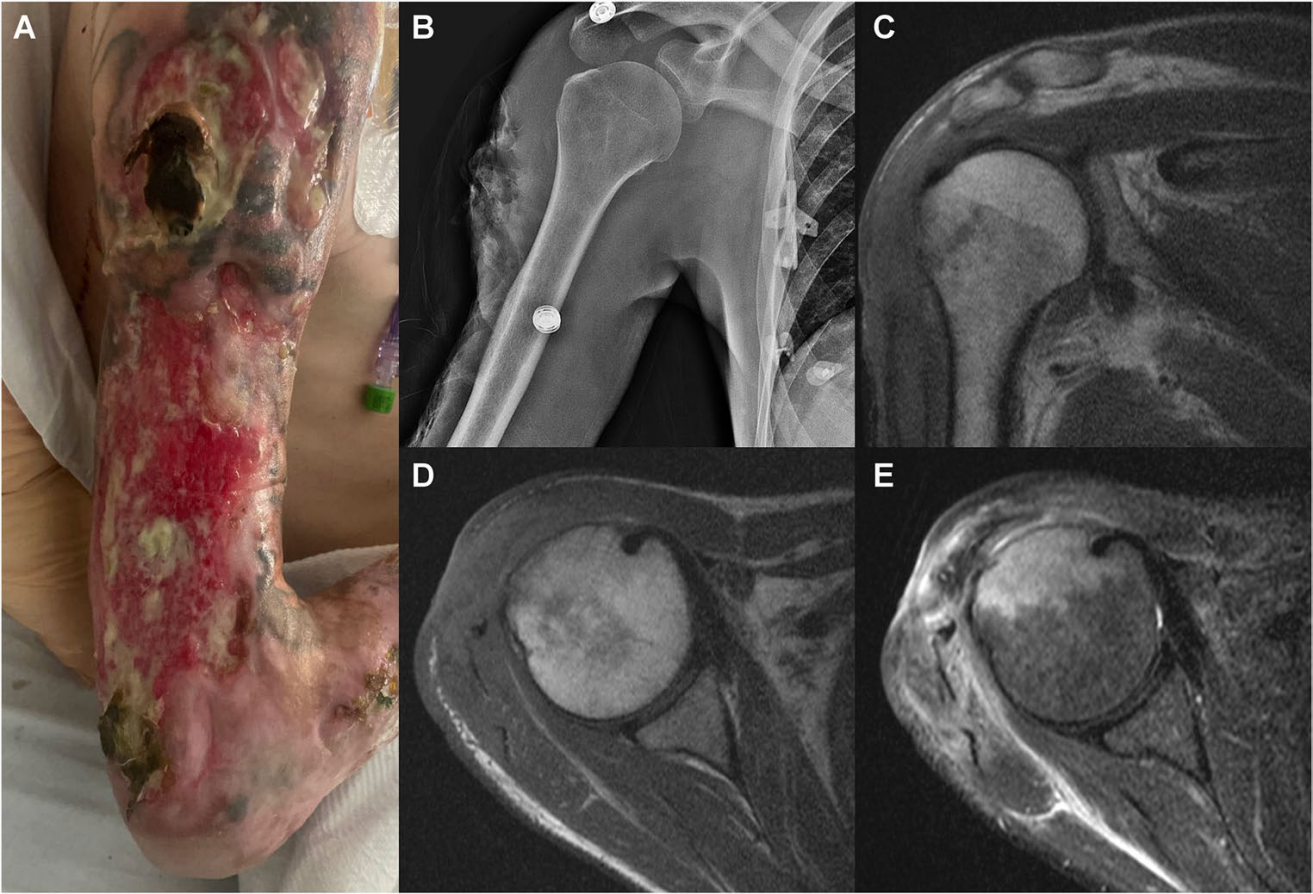
38-year-old man with an extensive xylazine-associated soft-tissue wound involving the right shoulder (**A**). (**B**) Radiograph demonstrates a soft-tissue defect and ulceration along the lateral shoulder and proximal upper arm. Coronal T1-weighted (**C**), axial T1-weighted (**D**), and axial short-τ inversion-recovery (STIR) (**E**) MR images demonstrate T1-hypointense marrow replacement involving the greater tuberosity, with a larger surrounding zone of marrow edema, consistent with acute osteomyelitis with adjacent reactive osteitis

True osseous infection was identified in a minority of patients (Table 2). When present, osteomyelitis typically occurred adjacent to chronic wounds, often in the setting of surrounding soft-tissue gas or exposed bone. On MRI, osteomyelitis demonstrated characteristic T1-hypointense marrow replacement with associated marrow edema and contrast enhancement extending from the cortical surface into the medullary cavity (Fig. 5). Cortical destruction, pathological fracture, and exposed bone were observed but were rare (Supplementary Fig. 8). In a subset of patients, imaging findings were consistent with chronic osteomyelitis, including sequestra and sinus tracts extending from soft-tissue wounds to intramedullary cystic lesions with sclerotic rims, compatible with intraosseous (Brodie) abscesses (Supplementary Fig. 7).

Despite the severity of the overlying soft-tissue injury, most patients did not demonstrate imaging evidence of osteomyelitis. Instead, xylazine-associated wounds most commonly demonstrated extensive superficial necrosis, long-segment subcutaneous fat obliteration, deep fascial and muscular involvement with evolving muscular hypertrophy, followed by progressive muscle loss and striking periosteal reaction that frequently occurred without destructive osseous changes. These findings underscore the importance of distinguishing reactive periostitis from true contiguous osseous infection.

## Discussion

Our study demonstrates that xylazine-associated wounds produce a distinctive pattern of musculoskeletal injury characterized by extensive soft-tissue necrosis, long-segment subcutaneous fat obliteration, deep fascial and muscular involvement, and prominent periosteal reaction that frequently occurs without destructive osseous change. To our knowledge, this represents one of the largest imaging-based characterizations of xylazine-associated wounds in patients who inject drugs. As xylazine continues to proliferate within the illicit drug supply, particularly in urban centers with high rates of injection drug use, radiologists and frontline clinicians must recognize the imaging patterns associated with its tissue complications.

Our findings support prior clinical observations that xylazine-associated wounds differ substantially from typical soft-tissue infections in both appearance and pathophysiology [11, 17]. In our cohort, these injuries frequently demonstrated extensive non-purulent ulcerative necrosis with variable depth and distribution, and the severity of the soft-tissue injury was often disproportionate to the degree of systemic inflammation or bone infection. A defining feature was the pattern of tissue destruction, which frequently extended transmurally from superficial layers to deeper structures rather than propagating longitudinally along fascial planes. In some cases, this transmural injury was associated with replacement of normal fascial planes by dense fibrinoid or inflammatory tissue, which may increase resistance to longitudinal spread and help explain the localized but deeply penetrating pattern of injury observed on imaging. When gas was present, it typically remained localized near the injection site or ulcer bed rather than tracking along fascial planes, in contrast to conventional musculoskeletal infections, in which gas and purulent material commonly spread along fascial planes. The underlying pathophysiology likely involves a combination of direct tissue toxicity, vasoconstriction-related ischemia, and repeated injection-related trauma, findings that align with reports describing xylazine’s vasoconstrictive and cytotoxic effects [12, 18]. Supporting this hypothesis, recent studies have demonstrated that xylazine can act directly on epithelial cells via the kappa opioid receptor, inducing cellular contraction and disrupting keratinocyte intercellular junctions [19, 20]. This mechanism provides a potential molecular explanation for the progressive tissue necrosis and structural disruption observed on imaging in affected patients.

Longitudinal imaging in several patients demonstrated progressive enlargement of soft-tissue lesions and evolving osseous changes over time, raising the possibility of a cumulative or dose-dependent relationship between repeated xylazine exposure and wound progression. Radiographs, the most frequently obtained imaging modality in our cohort, were often sufficient to demonstrate hallmark imaging features, including soft-tissue gas, distortion of deep soft-tissue planes, periosteal reaction, and cortical irregularity. Cross-sectional imaging with CT and MRI provided complementary information by delineating the full extent of soft-tissue involvement, identifying devitalized tissue, and assessing potential osseous complications. These modalities were particularly useful for evaluating deep fascial and muscular involvement, characterizing abscess formation, and distinguishing reactive osseous changes from true bone infection.

When bone abnormalities were present, exuberant periostitis and reactive osteitis were the predominant findings, whereas acute and chronic osteomyelitis occurred in a minority of patients. A key clinical implication of these findings is that an extensive periosteal reaction in this setting should not be automatically interpreted as osteomyelitis. In our cohort, periosteal new bone formation frequently occurred without destructive osseous change, marrow replacement, or other imaging features of contiguous infection. Misinterpretation of these findings may lead to overdiagnosis of osteomyelitis and potentially unnecessary interventions. In this vulnerable population, overdiagnosis carries important downstream consequences, including prolonged antibiotic therapy, increased risk of multidrug-resistant infections, and escalation to limb amputation [21]. Recognizing the distinction between reactive periostitis and true osseous infection is therefore essential for guiding appropriate clinical management.

This study has several limitations. Its retrospective design introduces the possibility of selection bias, as only patients with documented xylazine exposure who underwent imaging were included. Consequently, the true prevalence of xylazine-associated musculoskeletal complications is likely underestimated, particularly for early or mild cases that may not undergo imaging evaluation. In addition, clinical presentations and imaging time points varied among patients, limiting our ability to formally model the temporal progression of these lesions. A formal comparison of diagnostic performance between radiographs and CT was not performed, as not all patients underwent both modalities; this remains an important area for future investigation. Despite these limitations, our findings have important clinical implications. Given the high burden of complications and the frequent discharge against medical advice observed in this population, optimized radiologic evaluation at the time of initial presentation may improve diagnosis, triage, and management. As the public health impact of xylazine continues to expand, collaboration among radiologists, emergency physicians, infectious disease specialists, surgeons, and addiction medicine providers will be essential to improving outcomes.

In conclusion, xylazine-associated wounds represent a growing source of morbidity among people who inject drugs, frequently producing severe soft-tissue injury with characteristic imaging features and relatively infrequent osseous infection. Radiologic assessment plays a central role in diagnosis, disease characterization, and management. Recognition of these patterns is critical to avoid overdiagnosis of osteomyelitis and to guide appropriate patient care.

## Supplementary Information

Below is the link to the electronic supplementary material.

Supplementary File 1 (DOCX 2.23 MB)

## Data Availability

All data supporting the findings of this study are available within the paper and its Supplementary Information.
