# Supplementary material for: Musculoskeletal imaging findings of xylazine-associated wounds
Source: Emerg Radiol. 2026 Apr 28;33(4):755–63. doi: 10.1007/s10140-026-02480-4 (PMC13433553; doi:10.1007/s10140-026-02480-4)
Supplement: Supplementary file 1 [file 10140_2026_2480_MOESM1_ESM.docx]

**SUPPLEMENTARY FIGURES**

**Supplementary Figure 1**. (**A, B**) Right wrist ultrasound in a 46-year-old man demonstrating marked cutaneous thickening, subcutaneous edema, and hyperemia consistent with cellulitis. (**C, D**) Left wrist ultrasound in a 48-year-old man demonstrating a complex heterogeneous fluid collection with surrounding hyperemia consistent with a developing abscess. Note the undulation of the deep soft-tissue contour.

**Supplementary Figure 2. Longitudinal multimodality imaging of xylazine-associated lower-extremity wounds demonstrating progressive periostitis and eventual osteomyelitis.** Representative clinical photographs, radiographs, CT, and MRI from a 35-year-old man with recreational xylazine injection into the right lower extremity obtained at baseline and serial follow-up over 42 months. Not all imaging modalities were available at each time point, and the patient underwent multiple antibiotic courses and surgical debridements during follow-up. T2-weighted MR sequences included axial short τ inversion-recovery (STIR) or fat-saturated (FS) sequences, as indicated at each time point. At baseline, large anteromedial calf ulcers with diffuse subcutaneous edema and subtle tibial periosteal reaction are present on CT and MRI, compatible with osteitis without imaging evidence of osteomyelitis. At 3 months, the wounds have progressed, with new periosteal reaction and possible cortical erosion on radiographs and CT. MRI demonstrates skin defects extending to bone with diffuse muscular edema but no definite marrow replacement; acute osteomyelitis was diagnosed clinically based on exposed bone. At 5 months, radiographs and MRI demonstrate extensive periosteal reaction and cortical irregularity with small erosions and minimal marrow edema, compatible with acute-on-chronic osteitis and associated myositis. At 24 months, persistent large soft-tissue defects with long-segment tibial periostitis are present without abnormal marrow signal, favoring chronic reactive or infectious periostitis without definite osteomyelitis. At 32 months, progressive ulceration with cortical thickening and new cortical erosion is present, consistent with chronic periostitis with associated infectious myositis. At 42 months, radiographs, CT, and MRI demonstrate extensive destructive osteomyelitis of the mid-to-distal tibial shaft with circumferential periostitis, cortical erosions, mild medullary edema, and diffuse infectious myositis.

**Supplementary Figure 3. Progressive forearm wound with evolution to acute osteomyelitis in xylazine-associated injection injury.** Clinical photographs and corresponding radiographs from a 32-year-old woman with a large right forearm wound related to repeated recreational xylazine injection at initial emergency department presentation and at 9-month follow-up. At baseline, clinical photographs demonstrate a large, ulcerated forearm wound with marked surrounding edema and soft-tissue swelling. Radiographs demonstrate periosteal reaction involving the proximal to mid ulnar shaft without cortical destruction. At 9 months, there is marked interval enlargement of the forearm wound with extension to the proximal ulnar diaphysis and exposed bone. Radiographs demonstrate marked progression of lamellated periosteal reaction along the ulnar diaphysis, compatible with acute osteomyelitis in the setting of an open wound. The patient subsequently underwent transhumeral amputation.

**Supplementary Figure 4. Septic bursitis.** (**A**) A 33-year-old man presented with multiple wounds of the left upper extremity and diffuse erythema suggestive of cellulitis. (**B**) Elbow radiographs demonstrate soft-tissue swelling without osseous abnormalities to indicate osteomyelitis. (**C**) Sagittal (left) and axial (right) CT images acquired with soft-tissue (top) and bone (bottom) reconstruction kernels, displayed using corresponding window settings, demonstrate skin thickening and subcutaneous edema with a focal fluid collection overlying the olecranon, consistent with olecranon bursitis. The fluid collection was subsequently drained, with cultures positive for polymicrobial infection. The patient was discharged on antibiotic therapy.

**Supplementary Figure 5. Infected tendinitis and tendosynovitis.** (**A**) Skin wound in a 32-year-old woman with repeated xylazine injection involving the right lower extremity. (**B**) Contrast-enhanced CT performed on admission demonstrates diffuse soft-tissue swelling above the ankle extending to the dorsum of the foot. A small abscess (yellow arrow) abuts the anterior tibialis tendon (white arrow). The tendon is enlarged and heterogeneous at the level of the ankle, suggesting infected tendinitis with associated mild tenosynovitis, evidenced by a small amount of loculated fluid within the tendon sheath. No imaging evidence of osteomyelitis is present. The patient subsequently left against medical advice.

**Supplementary Figure 6.** **Soft tissue abscess.** Transverse (**A**) and coronal (**B**) CT images of a 37-year-old woman demonstrate swelling of the sternocleidomastoid muscle with bilateral intramuscular abscesses (yellow arrows) corresponding to sites of repeated xylazine injection. Surrounding intramuscular enhancement is present, consistent with myositis, with associated reactive cervical lymphadenopathy.

**Supplementary Figure 7. Brodie abscess with acute-on-chronic osteomyelitis.** (**A**) A 35-year-old woman presented to the emergency department with a large soft-tissue wound of the left lower leg in the setting of repeated xylazine injections. Radiographs demonstrate marked cortical and periosteal thickening with extensive superimposed lucencies along the medial and anterior aspects of the mid tibial shaft (yellow arrow), suggestive of acute-on-chronic osteomyelitis. (**B**) Contrast-enhanced CT of the ipsilateral lower leg demonstrates periosteal reaction, cortical thickening, and cortical erosion. Axial images reveal two small Brodie abscesses within the tibial shaft (yellow arrows) with associated sinus tract formation. (**C**) Axial follow-up CT images obtained 6 months later demonstrate interval progression of the abscesses. (**D**) MRI at the time of initial presentation demonstrates a large ulceration along the anteromedial tibial shaft with marked cortical thickening, cortical destruction, heterogeneous low T1 marrow signal, and high T2 signal, consistent with acute-on-chronic osteomyelitis involving the tibial shaft. The fibula demonstrates periosteal reaction without T1 marrow signal abnormality and mild patchy increased T2 signal, suggestive of reactive osteitis.

**Supplementary Figure 8. Pathologic fracture.** (**A**) A 42-year-old man presented with extensive soft-tissue wounds of the right arm following repeated injections of fentanyl and xylazine at this site. Radiographs demonstrate an exposed fracture of the mid-ulnar diaphysis with periosteal reaction involving the mid to distal radius and ulna, suspicious for osteomyelitis. The patient subsequently left against medical advice. (**B**) At repeat presentation to the emergency department three months later, the right arm wounds had progressed. Radiographs demonstrate open fractures of the mid ulnar and radial shafts with displacement and overriding of the radial shaft, extensive periosteal reaction, cortical erosions, and osseous remodeling, findings consistent with pathologic fractures secondary to osteomyelitis. The patient subsequently underwent proximal forearm transradial amputation.
